# Supplementary figures and images for: A molecular insight into the lipid changes of pig Longissimus thoracis muscle following dietary supplementation with functional ingredients
Source: PLoS One. 2022 Mar 24;17(3):e0264953. doi: 10.1371/journal.pone.0264953 (PMC8947141; doi:10.1371/journal.pone.0264953)

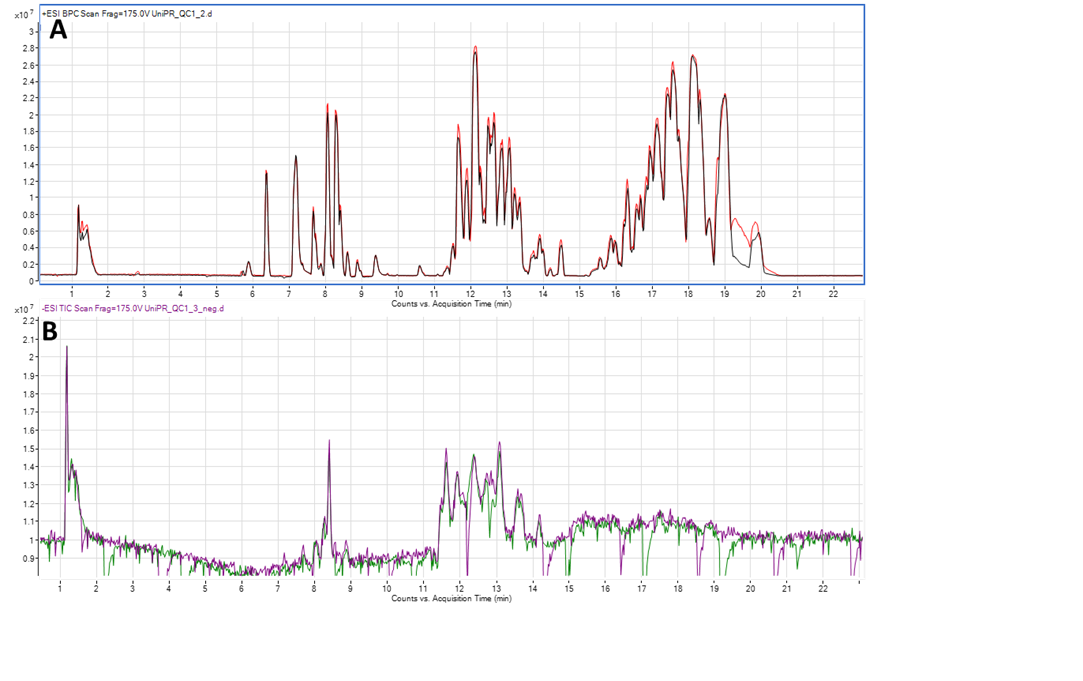

Supplement: S1 Fig — Ultra-high-performance liquid chromatography coupled to high resolution mass spectrometry base peak chromatograms of meat sample extracts obtained using positive (A) and negative (B) ionization modes. (TIF) [file pone.0264953.s001.tif]

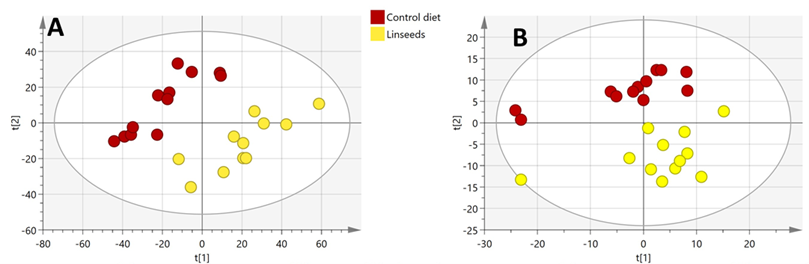

Supplement: S2 Fig — Scatter score plots for PCA models built from positive (A) and negative (B) ionization modes. (TIF) [file pone.0264953.s002.tif]

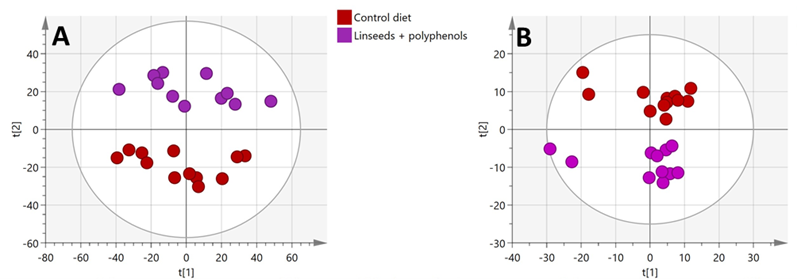

Supplement: S3 Fig — Scatter score plots for PCA models built from positive (A) and negative (B) ionization modes. (TIF) [file pone.0264953.s003.tif]

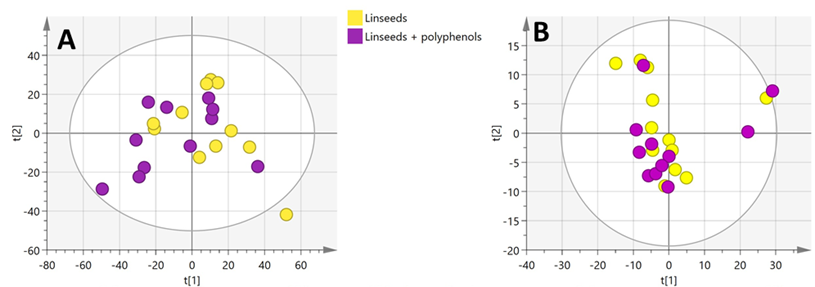

Supplement: S4 Fig — Scatter score plots for PCA models built from positive (A) and negative (B) ionization modes. (TIF) [file pone.0264953.s004.tif]

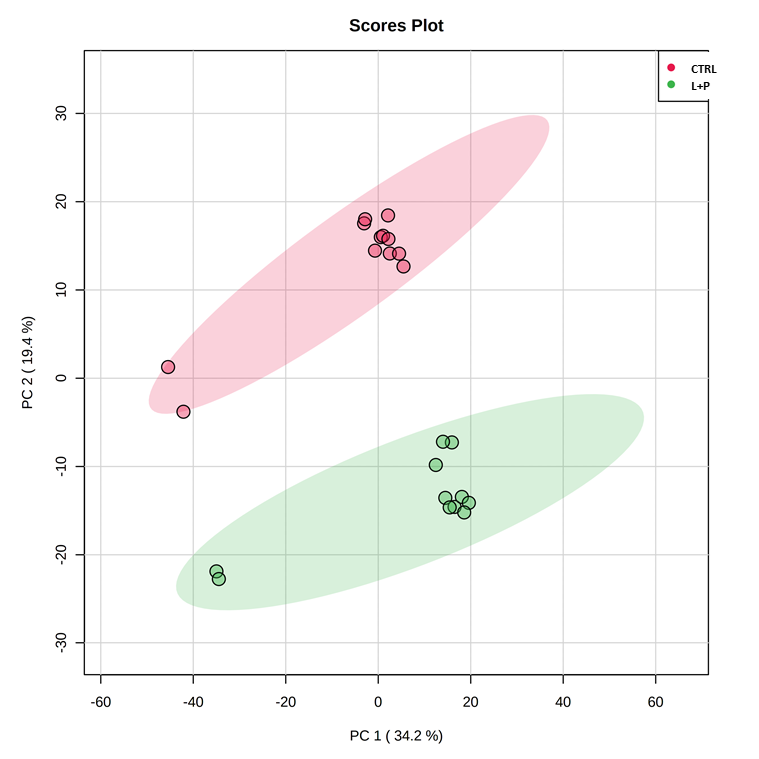

Supplement: S5 Fig — (TIF) [file pone.0264953.s005.tif]

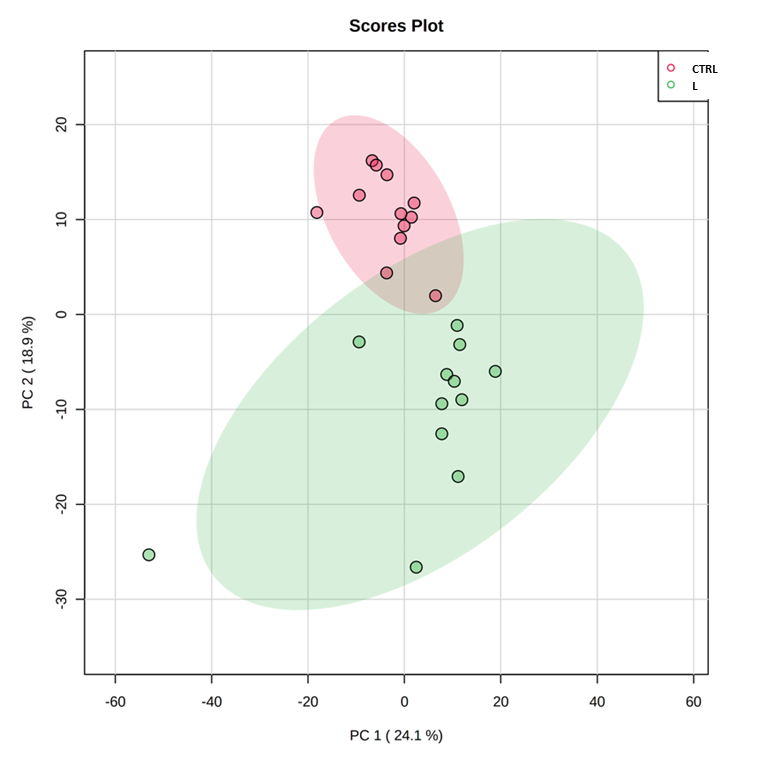

Supplement: S6 Fig — (TIF) [file pone.0264953.s006.tif]
