# Supplementary material for: A molecular insight into the lipid changes of pig Longissimus thoracis muscle following dietary supplementation with functional ingredients
Source: PLoS One. 2022 Mar 24;17(3):e0264953. doi: 10.1371/journal.pone.0264953 (PMC8947141; doi:10.1371/journal.pone.0264953)
Supplement: Attachment — Submitted filename: Response to decision letter.doc [file pone.0264953.s020.doc]

Parma, 13.12.2021

Dear Editor,

This is to express our thanks for a comprehensive review of our manuscript N° PONE-D-21-25228. On behalf of my co-authors, I am submitting the revised version of the manuscript “***A molecular insight into the lipid remodeling of pig Longissimus thoracis muscle following dietary supplementation with functional ingredients***” written by Rocchetti et al. to be considered for publication in your journal “PlosOne”.

We have revised it carefully and incorporated reviewer’ critical comments/suggestions into the attached revision. We acknowledge the reviewers for their comments because enable us to improve the quality of the manuscript.

All our answers/scientific opinions are summarized in the paragraphs below and changes in the manuscript are highlighted in red and using the track changes mode in MS Word

Thank you for your kind attention and interest.

With compliments,

Dr. Laura Righetti and Dr. Martina Zappaterra

Reviewers' comments:

Reviewer's Responses to Questions

**Comments to the Author**

1. Is the manuscript technically sound, and do the data support the conclusions?

The manuscript must describe a technically sound piece of scientific research with data that supports the conclusions. Experiments must have been conducted rigorously, with appropriate controls, replication, and sample sizes. The conclusions must be drawn appropriately based on the data presented.

Reviewer #1: Partly

2. Has the statistical analysis been performed appropriately and rigorously?

Reviewer #1: I Don't Know

3. Have the authors made all data underlying the findings in their manuscript fully available?

The [PLOS Data policy](https://eur01.safelinks.protection.outlook.com/?url=http%3A%2F%2Fwww.plosone.org%2Fstatic%2Fpolicies.action%23sharing&data=04|01|laura.righetti@unipr.it|c9d223a82ca34c44365908d99e44587e|bb064bc5b7a841ecbabed7beb3faeb1c|0|0|637714839374799299|Unknown|TWFpbGZsb3d8eyJWIjoiMC4wLjAwMDAiLCJQIjoiV2luMzIiLCJBTiI6Ik1haWwiLCJXVCI6Mn0%3D|3000&sdata=T36Jw8%2FV5vC8rUWe%2B0wYxZAWNrT5jNqYONrthlmbA3w%3D&reserved=0) requires authors to make all data underlying the findings described in their manuscript fully available without restriction, with rare exception (please refer to the Data Availability Statement in the manuscript PDF file). The data should be provided as part of the manuscript or its supporting information, or deposited to a public repository. For example, in addition to summary statistics, the data points behind means, medians and variance measures should be available. If there are restrictions on publicly sharing data—e.g. participant privacy or use of data from a third party—those must be specified.

Reviewer #1: Yes

4. Is the manuscript presented in an intelligible fashion and written in standard English?

PLOS ONE does not copyedit accepted manuscripts, so the language in submitted articles must be clear, correct, and unambiguous. Any typographical or grammatical errors should be corrected at revision, so please note any specific errors here.

Reviewer #1: Yes

5. Review Comments to the Author

Please use the space provided to explain your answers to the questions above. You may also include additional comments for the author, including concerns about dual publication, research ethics, or publication ethics. (Please upload your review as an attachment if it exceeds 20,000 characters)

Reviewer #1: General Comments:

This manuscript presents original results from a study evaluating longissimus muscle lipidome and transcriptome in swine fed diets with a different fat profile. Part of the data has already been published in two companion papers. The study is scientifically sound and has a good number of experimental units, but I think it is missing information about the carcass and meat traits. I mean, was there a difference in intramuscular fat?
AU: We thank the reviewer for the time spent revising the manuscript and the useful suggestions. We have revised the manuscript following the comments and we hope the revised manuscript could be clearer now. The results reported in this manuscript originate from a research project that involved, in addition to the Authors of the present paper, other research teams with different expertise and involved in various research fields. The project was conceived to meet the goal of integrating diverse competences and skills, in a holistic approach. Data for meat and carcass quality were published in the paper by Belmonte et al. (2021; Effects of high linolenic acid diet supplemented with synthetic or natural antioxidant mix on live performance, carcass traits, meat quality and fatty acid composition of Longissimus thoracis et lumborum muscle of medium-heavy pigs. Italian Journal of Food Science, 33 (2): 117–128, doi: 10.15586/ijfs.v33i2.2005). This paper has now been added in the revised version of the manuscript as reference 21.

The authors should review the proposed title. They did not evaluate lipid remodeling (i.e., synthesis and degradation). You have a transcriptomic snapshot and a lipidomic snapshot, but you did not evaluate it over time.

AU: We agree with the reviewer about this important remark. Therefore, we have modified the title, accordingly, in: "A molecular insight into the lipid changes of pig *Longissimus thoracis* muscle following dietary supplementation with functional ingredients"

The language is good, although a couple of words could be replaced to improve clarity (please see the specific comments below).

AU: Thank you for the feedback. We have re-checked and improved the language in the revised version of the manuscript, accordingly.

General Questions:
1. Would authors consider renaming the treatments? It could make the text more informative [e.g., instead of D1 => CTRL; D2 = L; D3 => L+P].

AU: We would like to thank the reviewer for the suggestion aimed at improving the overall clarity of the text. We have modified the names of the treatments, accordingly.

2. Why do you choose to study meat fatty acid profile instead of subcutaneous FA profile? Maybe pork bellies FA.

AU: The reason is that loin is one of the most consumed cuts of meat in countries where pork is part of the human diet. On the contrary, subcutaneous fat is often discarded by consumers, or is used for local and typical pig meat productions that are not consumed with high frequency (such as Colonnata lard obtained from the Cinta Senese Italian autochthonous pig breed ). Therefore, for the present study, we opted for the *Longissimus thoracis* muscle.

3. Was there a difference in intramuscular fat among treatments?

AU: Carcass and meat quality data were reported in the manuscript by Belmonte et al. (2021; Effects of high linolenic acid diet supplemented with synthetic or natural antioxidant mix on live performance, carcass traits, meat quality and fatty acid composition of Longissimus thoracis et lumborum muscle of medium-heavy pigs. Italian Journal of Food Science, 33 (2): 117–128, doi: 10.15586/ijfs.v33i2.2005). Overall, no difference was noticed among diets for the total fatty acids (CTRL = 679 ± 64 mg/g of lipids; L = 667 ± 79 mg/g of lipids; L+P=712 ± 48 mg/g of lipids; *P*-value of the comparison= 0.41) and total lipids (CTRL = 1.65 ± 0.48 %; L = 1.55 ± 0.51 %; L+P=1.78 ± 0.53 %; *P*-value of the comparison= 0.28) of the *Longissimus thoracis* muscle.

4. I understand the contract D1 vs. D2, but why are you contrasting D1 vs D3, but not D2 vs D3? I mean how can you separate what is PUFA effect and what is the effect of polyphenols?

AU: We understand the remark made by the reviewer. Indeed, in this work, we have initially observed by using unsupervised and supervised statistical approaches a certain similarity between the lipidomic profile of D2 (L) and D3 (L+P) diets when compared with the D1 (CTRL) diet. Therefore, also considering the restrictions of the software used to provide multivariate statistics (MetaboAnalyst), where multiple comparisons are not allowed to provide Volcano plot analysis and statistically significant marker compounds, we firstly decided to compare each experimental diet *vs* the control diet (that was a fixed effect) and then we used a Venn diagram to extrapolate the exclusive up-accumulated marker lipids for both D2 (L) and D3 (L+P) diets vs the D1 (CTRL) diet. However, considering the remark made by the reviewer and the possible interest of a food science and technology readership, we have added also the multivariate statistical approach for the comparison D2 (L) vs D3 (L+P) to discern the effect of polyphenols from grape skin and oregano extracts added to the feeding system. Please, consider that a comprehensive MS-based analysis of each feeding system has not been conducted, therefore further works are still needed to correlate the metabolomic and lipidomic profile of the feeding system with the final lipidomic profile of meat in terms of improving oxidative stability. Finally, please consider that, in this work, the focus was based on the potential correlations existing between the discriminant lipids (as outlined by lipidomics) vs CTRL diet and the discriminant genes (as outlined by transcriptomics).

5. The introduction should be edited adding the rationality of adding polyphenol to pigs diet and why add it to a diet with PUFA.

AU: Thank you for the important remark. we have added some additional sentences to explain the rationality of adding polyphenols to pigs’ diet based on PUFA.

6. Why did you run a supervised and unsupervised statistical model? I mean, shouldn’t you choose one only. If they show different results, which one are you going to consider?
AU: We would like to thank the reviewer for this important question. Indeed, our multivariate statistical approach is strictly following the state-of-the-art of untargeted metabolomics studies. In this regard, when performing untargeted metabolomics, both supervised and unsupervised models must be considered. Firstly, raw mass features are elaborated by using unsupervised statistical approaches (such as PCA or hierarchical clustering approaches) to detect sample clustering in the measured data and to provide an initial overview of the trend, including the determination of putative outliers. After data quality assessment check, supervised models, such as PLS-DA and/or OPLS-DA approaches are considered, to maximize differences between sample treatments and to highlight key variables and potential markers (such as VIP compounds or S-plot discriminant metabolites) of the trends observed. Besides, according to OPLS-DA approaches, the variation between the groups can be separated into predictive and orthogonal (i.e., ascribable to technical and biological variation) components. Therefore, if there are substantial differences in the metabolome, a clustering should be detected already in the unsupervised model and the maximized when using the supervised model.

Specific Comments:
L. 32- Is this quoted right? Does PUFA improve oxidative stability? If so, why do you have to add “natural antioxidants” as highlighted in L.33?

AU: We apologize for the misleading sentence that was incomplete in the previous version of the manuscript. PUFA can worsen the oxidative stability of the meat products and to avoid this problem antioxidant compounds are often supplemented in the diets integrated with PUFA.

The revised sentence is: " The most used strategies were based on changes of the nutritional profile by supplementing animal diets rich in polyunsaturated fatty acid (PUFA) with antioxidant compounds (such as vitamin E) to improve the oxidative stability of the meat (3)".

L. 37- I suggest replacing “determining the” by “leading to”
AU: Thank you for the suggestion. Amended.

L. 48- Is it forage a source of fatty acids in swine’s diet?

AU: We apologize for the misleading sentence. We fully agree with the Reviewer that forage is not used in pig feeding. We have deleted it accordingly.

L. 48- I suggest replacing “exploited” by “used”

AU: Thank you, done.

L. 51- Please, replace the word “react”. I don’t think it is what you want to say

AU: Thank you for the suggestion. We have replaced “react” with “respond”.

L. 51- Please review the citation “hormones8.”

AU: Sorry for the mistake. It was a typo. Done.

L. 58- What are referring to with “later” here.

AU: We agree with the Reviewer that this sentence was not clear. We have rephrased the initial part of the sentence replacing “the latter” with “those genes”.

L. 65- Replace “pig meat” with “pork”

AU: The term has been replaced throughout the manuscript, accordingly.

L. 93- 5% of what

AU: We have added “on wet basis”.

L. 99- “slaughter”

AU: Revised, accordingly.

L. 100- Please review across the manuscript the table and figure citations (e.g., L. 212, L.218). I think they should be capitalized

AU: Thank you for the suggestion. We have revised the tables and figures citations, accordingly.

L. 103- 105 what?

AU: Sorry, it was 150 kg. We have added the measure unit.

L. 104-105- Please, specify the exact sampling place

AU: The information has been added, accordingly. The revised sentence is now in lines 113-114. “At the end of the slaughter line and before carcasses were cooled down, two samples of the *Longissimus thoracis* *et lumborum* muscle were gathered from the left side of each carcass, on the back over the joint between last thoracic and first lumbar vertebrae”.

L. 108- Delete “meat”

AU: Deleted, accordingly.

L. 108- “handshaking” is this term right?
AU: We apologize for the misleading term. The sentence has been revised, accordingly.

L. 120- define “HRMS”

AU: High Resolution Mass Spectrometry (HRMS)

L. 153- “metabolomic” is this term right? (same for L.207)

AU: Sorry, it was a typo, we changed it with “metabolomics”.

L. 160- Why you did not evaluate D2vs. D3?

AU: The comparison D2 (L) vs D3 (L+P) is now provided in the revised version of the manuscript. Thank you for pointing it out.

L. 162- I don’t think it is clear which “same software” you are talking about

AU: We have added the name of the software (MetaboAnalyst).

L. 178-179- Please add a reference for these methods.
AU: Thanks, agreed. The citation Shi et al. 2021 (doi: https://doi.org/10.1186/s13638-021-01910-w) has been added accordingly.

L. 198- At the beginning of the discussion, the authors should provide a little background on animal growth performance and carcass and meat quality traits. So, is this difference in lipidomic somehow affected by the pork fat percentage? I mean, we know that phospholipids have more PUFA than neutral lipids stored inside an adipocyte. So, is the fat percentage

AU: We agree with the reviewer that this part could be of interest for the reader and therefore we have added a part about that at the beginning of the results and discussion section.

L. 202- “lipid metabolites” is this term right?

AU: Sorry, it was a mistake, we changed it with “lipids”.

L. 230- What are these “derivatives”? did you mention it earlier in the text?

AU: We apologize for the misleading term. We referred to the different lipid classes and subclasses (as reported in supplementary material). The sentence has been revised, accordingly.

L. 291- Can you add a reference to support this sentence?
AU: The citation Matthews et al. 2000 (doi: doi:10.1017/S0007114500000817) has been added accordingly.

L. 298- Please add a reference

AU: Thank you for the useful suggestion. We have added two references here and in the following sentence (e.g., Raphael and Sordillo 2013, doi:10.3390/ijms141021167; and Domínguez et al. 2019, doi:10.3390/antiox8100429).

L. 307-309- Did you evaluate fat oxidation? Like TBARS

AU: The paper by Belmonte et al. (2021; doi: 10.15586/ijfs.v33i2.2005) reported the results of the lipid stability in raw and cooked samples coming from the same muscle (*Longissimus thoracis*) and the same animals used in the present study. Belmonte et al. found that the considered diets did not cause differences in the oxidative stability during meat storage and cooking of the *Longissimus thoracis* samples.

L. 312-314- Please add a reference

AU: Done.

L. 326- what is “CTR”

AU: We referred to the control diet. We have revised the term with "CTRL" according to the reviewer's suggestion.

L. 333- add a “respectively” after “annotations”

AU: Done.

L. 339- which “figure” are you talking about?
AU: We agree with the Reviewer that this part was not clear. We were referring to Figure S6, which was cited in the previous sentence. We have replaced “the figure” with “this figure”.

L. 354-358- I am not sure if your statistical comparison allows you to affirm this. In my opinion, you should contrast D2 and D3.

AU: We have modified the sentence highlighted by the reviewer and provided in the revised version of the manuscript the contrast D2 (L) vs D3 (L+P).

L. 364- what is “control group”?
AU: Sorry, it was a mistake. We were referring to the first diet (CTRL).

L. 369- which “figure” are you talking about?

AU: Sorry. We were referring to Figure 4, which was cited in the previous sentence. We have replaced “the figure” with “this figure”.

L. 388-391- I don’t think you can infer that PUFA is promoting lipid remodeling, based on your data. Again, lipid remodeling is a change over time, and you evaluated only one timepoint.

AU: We accept the reviewer's remark. Indeed, we have not evaluated the lipid remodeling over time, but we are describing only a snapshot of the lipid changes induced from the two experimental diets (i.e., L and L+P) when compared with the control diet (CTRL). Therefore, according to the reviewer's remark, we have modified the title of this manuscript.

L. 394-397- Please add a reference

AU: Done.

L. 410-412- See comment for L.388-391

AU: We agree with the reviewer, and we have modified this sentence.

Table 4- Which lipids are these genes correlated to? Total lipids in meat?

AU: No, we are not considering total lipids in this manuscript. These genes are correlated with several discriminant lipids identified in this paper by using the lipidomic approach and reported in the supplementary Table S10.

L. 540-541- Please rephrase this sentence.

AU: Thank you for the useful suggestion. We agree that this sentence was not clearly phrased. The revised sentence is as follows: “According to the same review, the different DG species can be involved in different pathways in the regulation of cell metabolism, growth and apoptosis; however full knowledge of how DG species regulate cell metabolism, growth and apoptosis is still lacking (43).”

L. 562- I don’t think ECM is considered “cytoskeleton”. Please review this sentence.

AU: We fully agree with the Reviewer. We have amended this sentence as follows: In a study by Mariman and Wang (46), conducted on MSC-derived adipogenic ECM, several genes have been related to adipogenic differentiation and ECM development, confirming an association between the expression of these DEGs and an effect on the cellular structure and integrity.”

L. 571-572- Please add a reference

AU: Thank you for the suggestion. We have added the following citation here: Breslow and Weissman, 2010, doi:10.1016/j.molcel.2010.10.005.

L. 583- I don’t think this approach is a good one to study cell membrane remodeling.

AU: We understand the reviewer's remark. We have modified the title of the manuscript together with some key sentences about cell membrane remodeling throughout the manuscript to avoid misunderstandings. We are now talking about lipidomic changes induced by two different experimental diets when compared with a control diet, and the potential correlations existing between discriminant lipids and genes.

L. 585- Please rephrase it replacing “each experimental diet”. Here at the conclusion, you should show in a few sentences the effect of PUFA and polyphenol on lipidomic. Avoid using D1, D2, or D3.

AU: Thank you for the remark. The Conclusions section has been revised, accordingly.

L. 585- What do you mean by “restricted”?

AU: We apologize with the reviewer, but we were unable to find the term "restricted" at line 585.

Figure 1- I think you should relabel the axes. Maybe put PCA1 (% of the variance explained). So, here you are not using a consistent treatment label. I mean, there is no D1, D2, and D3.

AU: Figure 1A and Figure 1B has been revised and the treatments have been renamed according to reviewer’s suggestion.

Figure 4- how were these data generated? I mean did you contrast D2 and D3? A lipid that is more accumulated in D2 and D3 compared to D1 may not have a similar accumulation if you compare D2 against D3.

AU: We would like to thank the reviewer for the important remark. Figure was generated by using a Venn diagram, thus evaluating both the common and exclusive UP-accumulated compounds for the contrast D3 (L+P) *vs* D1 (CTRL) and D2 (L) *vs* D1 (CTRL). As reported in the previous answer, we have initially observed by using unsupervised and supervised statistical approaches a certain similarity between the lipidomic profile of D2 (L) and D3 (L+P) diets when compared with the D1 (CTRL) diet. Therefore, also considering the restrictions of the software used to provide multivariate statistics (MetaboAnalyst), where multiple comparisons are not allowed to provide Volcano plot analysis and statistically significant marker compounds, we firstly decided to compare each experimental diet *vs* the control diet (that was a fixed effect) and then we used the Venn diagram to extrapolate the exclusive up-accumulated marker lipids for both D2 (L) and D3 (L+P) diets vs the D1 (CTRL) diet. However, considering the remark made by the reviewer and the possible interest of a food science and technology readership, we have added also the multivariate statistical approach for the comparison D2 (L) vs D3 (L+P) to discern the effect of polyphenols from grape skin and oregano extracts added to the feeding system. The integrated lipidomic and transcriptomic analysis did not evidence metabolites entering with high weights in PC1. This result, therefore, indicates that L vs. L+P diets comparison did not lead to large differences in the lipidomic profile of the samples, and most of the differences observed in the transcriptome are related to genes that are not involved in lipid metabolism.

While revising your submission, please upload your figure files to the Preflight Analysis and Conversion Engine (PACE) digital diagnostic tool, <https://pacev2.apexcovantage.com/>. PACE helps ensure that figures meet PLOS requirements. To use PACE, you must first register as a user. Registration is free. Then, login and navigate to the UPLOAD tab, where you will find detailed instructions on how to use the tool. If you encounter any issues or have any questions when using PACE, please email PLOS at [figures@plos.org](mailto:figures@plos.org). Please note that Supporting Information files do not need this step.
